# Improving N-terminal protein annotation of *Plasmodium* species based on signal peptide prediction of orthologous proteins

**DOI:** 10.1186/1475-2875-11-375

**Published:** 2012-11-15

**Authors:** Armando de Menezes Neto, Denise A Alvarenga, Antônio M Rezende, Sarah S Resende, Ricardo de Souza Ribeiro, Cor JF Fontes, Luzia H Carvalho, Cristiana F Alves de Brito

**Affiliations:** 1 Laboratory of Malaria, Rene Rachou Research Institute, Fiocruz Minas, Belo Horizonte, Brazil; 2 Laboratory of Cellular and Molecular Parasitology, Rene Rachou Research Institute, Fiocruz Minas, Belo Horizonte, Brazil; 3 Universitary Hospital Julio Muller, Cuiabá, Mato Grosso, Brazil

**Keywords:** Signal peptide, Orthologous, Gene annotation, Malaria, Plasmodium

## Abstract

**Background:**

Signal peptide is one of the most important motifs involved in protein trafficking and it ultimately influences protein function. Considering the expected functional conservation among orthologs it was hypothesized that divergence in signal peptides within orthologous groups is mainly due to N-terminal protein sequence misannotation. Thus, discrepancies in signal peptide prediction of orthologous proteins were used to identify misannotated proteins in five *Plasmodium* species.

**Methods:**

Signal peptide (SignalP) and orthology (OrthoMCL) were combined in an innovative strategy to identify orthologous groups showing discrepancies in signal peptide prediction among their protein members (Mixed groups). In a comparative analysis, multiple alignments for each of these groups and gene models were visually inspected in search of misannotated proteins and, whenever possible, alternative gene models were proposed. Thresholds for signal peptide prediction parameters were also modified to reduce their impact as a possible source of discrepancy among orthologs. Validation of new gene models was based on RT-PCR (few examples) or on experimental evidence already published (ApiLoc).

**Results:**

The rate of misannotated proteins was significantly higher in *Mixed* groups than in *Positive* or *Negative* groups, corroborating the proposed hypothesis. A total of 478 proteins were reannotated and change of signal peptide prediction from negative to positive was the most common. Reannotations triggered the conversion of almost 50% of all *Mixed* groups, which were further reduced by optimization of signal peptide prediction parameters.

**Conclusions:**

The methodological novelty proposed here combining orthology and signal peptide prediction proved to be an effective strategy for the identification of proteins showing wrongly N-terminal annotated sequences, and it might have an important impact in the available data for genome-wide searching of potential vaccine and drug targets and proteins involved in host/parasite interactions, as demonstrated for five *Plasmodium* species.

## Background

Malaria is the most devastating parasitic disease in the world. The disease affects more than 216 million people and kills nearly 655,000 people every year [1]. More than forty percent of the world’s population lives at risk of infection [2]. Parasite resistance to available chemotherapy drugs and also vector resistance to insecticides are increasing and spreading around the world, which impacts disease control [3,4]. The persistent huge socioeconomic impact of the disease and reports of resurgence in African countries show that, despite the control efforts, malaria is still a global health challenge [5]. Hence, new strategies to control malaria are essential to combat, eliminate or even eradicate the disease [6].

In recent years, the sequencing of genomes, transcriptomes and proteomes and their related high-throughput analyses have become major strategies for unraveling the detailed aspects of *Plasmodium* biology and the interactions between the parasite and its vertebrate and invertebrate hosts [7,8]. Genome sequence data is available for at least eight *Plasmodium* species, providing opportunities for many groups to join the renaissance in malaria research and translate this massive amount of data into commercially available new drugs and anti-malarial vaccines, which are still promises of the genomic era [3,9].

The first step in genome-wide identification of new drug targets or vaccine candidates is mainly based on identification of molecules in the interface between parasite and hosts or members of unique biochemical pathways in the parasite through *in silico* strategies, and these analyses are highly dependent on the accuracy of genome annotations. Massive-scale sequencing has considerably improved the annotation strategies, particularly the process of identifying coding sequences (CDS) [10]. However, gene annotation is still far from trivial, especially in eukaryotic genomes, and one of the most difficult tasks is the identification of the initial methionine and intron/exons boundaries [11].

Homology reflects the evolutionary history of genes and, after the recent expansion of genomics, has reemerged as one of the key concepts of evolutionary biology. Orthologs are genes derived from a single ancestral gene in the last common ancestor of the species being compared, whereas paralogs are genes related via duplication events [12]. Orthology is the basis of any comparative genetic analysis, because orthologs tend to retain equivalent molecular and biological functions [13], justifying its use in interspecific comparative analyses to assist in gene annotation, by exploring evolutionary gene histories, conservation, variability of molecular sequences and functional characterizations [14].

Protein trafficking is essential for all organisms and this process is primarily governed by intrinsic signals found in protein sequences. The best-known and studied transport motif is the signal peptide, usually located in the N-terminal end of proteins that are translocated across the plasma membrane (prokaryotes) or the endoplasmic reticulum membrane (eukaryotes) [15-17]. Signal peptides play an indirect role on the biological function of proteins in the sense that they help determine the subcellular environment a given protein will be available for interactions [18]. Since function is usually conserved among orthologous proteins, it was hypothesized that subcellular localization and, consequently, signal peptide status are expected to behave accordingly. Divergences among orthologs could be explained by (*i*) misannotation of protein sequences; (*ii*) limitations of the methodologies used to predict signal peptides or to assign orthology relationships; or (*iii*) true biological divergence resulting from singular evolutionary history of that gene in each species.

In order to study the source of divergences among *Plasmodium* proteins, an innovative yet computationally simple strategy was devised, in which signal peptide predictions and orthology were combined. This strategy helped to determine the prevalence of N-terminal sequence misannotations among *Plasmodium* proteins and, more importantly, it guided the process of revision of misannotated proteins, therefore improving the available information on protein sorting for the genus.

## Methods

### Combining orthology and signal peptide prediction: classification and selection of orthologous groups

Amino acid sequences for all predicted proteins from *Plasmodium vivax*, *Plasmodium knowlesi*, *Plasmodium falciparum*, *Plasmodium berghei* and *Plasmodium yoelii* were obtained from PlasmoDB (version 7.1) [19] along with information on their clustering within orthologous groups, according to OrthoMCL (version 4) [20]. *Plasmodium chabaudi* sequences were not included in this study due to systematic annotation inconsistencies in its dataset from PlasmoDB (version 7.1). Protein sequences were then submitted to signal peptide prediction using SignalP 3.0 [21] following the default settings from PlasmoDB. SignalP 3.0 employs two methods for the prediction of signal peptides, SignalP-NN and SignalP-HMM. The former runs two artificial Neural Networks and outputs five scores (S-score, C-score, Y-score, Mean-S and D-score) that vary gradually from 0 to 1 and have different thresholds. The D-score was proven to be the best discriminating parameter for SP prediction. SignalP-HMM uses a Hidden Markov Model and also gives out a score, termed Signal peptide probability (HMM probability), which also varies from 0 to 1. During a run of SignalP 3.0, SignalP-NN and SignalP-HMM can be used either concurrently or separately. Standard settings for SignalP 3.0 state that a given protein is positive if its D-score or its HMM probability are equal or higher than 0.43 and 0.5, respectively. However, the criteria for considering a positive prediction in PlasmoDB are different from the software's stand-alone version. In the default configurations of PlasmoDB, both D-score and HMM probability were kept as single parameters with thresholds of 0.5 and a third parameter was set by combining the remaining four scores (S-score, C-score, Y-score, Mean-S) from SignalP-NN. Each of the four scores was given a value of 1, if its threshold was met or 0 otherwise, the third parameter is the sum of values from each of the four scores, therefore varying from 0 to 4 in a unitary scale, and its preset threshold is 3. In PlasmoDB, a signal peptide prediction is considered positive if any of the three parameters is above the established cutoffs. By default SignalP 3.0 only considers the 70 amino acids on the N-terminal end of proteins for predicting signal peptides.

Orthology information (from OrthoMCLDB version 4), extracted from PlasmoDB (version 7.1), was used to cluster proteins into their respective orthologous groups. Signal peptide predictions (negative/positive) were analysed within orthologous groups and were used to classify groups into three classes: (*i*) *Positive* groups, in which all proteins have a positive signal peptide prediction; (*ii*) *Negative* groups, in which all proteins have negative signal peptide predictions; and (*iii*) *Mixed* groups, those presenting a mosaic of positive and negative signal peptide predictions among orthologous proteins. Groups containing two or more proteins from the same species (paralogous proteins) were excluded from inspection and reannotation (see Discussion section).

### Inspection of orthologous groups and reannotation of proteins

Each orthologous group was submitted to multiple global alignments of their proteins by MAFFT (version 6.717b) using default parameters [22]. Alignments for all *Mixed* groups were visualized using Jalview (version 2.6.1) [23] and manually inspected in search of putatively misannotations in protein sequences. Inspection was directed at the N-terminal end of proteins, approximately the first 100 amino acids, since SignalP was set to analyse the first 70 amino acids. A protein was considered putatively misannotated and was selected for reannotation when sequence inconsistencies (missing or protruding stretches of amino acids) or a clear lack of sequence conservation restricted to one or few proteins were observed, after comparative analyses among orthologs (see examples in Additional file 1). Nucleotide coding sequences, their upstream flanking regions and the coordinates for their original gene models were obtained, from PlasmoDB (version 7.1), for each selected protein and its orthologs as well. The original gene models were projected onto these nucleotide sequences and analysed with the Artemis software (release 12.0) [24]. Several features of the gene models such as number and size of exons, conservation of exon/intron junctions, and relative position of putative initial methionine to neighboring landmarks were subjected to a comparative analysis. Whenever possible, an alternative gene model, featuring the proposed exon boundaries of the CDS of reannotated protein, was proposed. It is important to note that signal peptide predictions were only used to classify groups, but were not used to guide the selection of which proteins should be reannotated, these were chosen based exclusively on manual inspections of multiple alignments.

After complete inspection (alignment and gene models) of all *Mixed* groups, they were further separated into three categories: (*i*) *No Misannotations*, where visual inspection did not detect any protein sequence that needed reannotation (Additional file 1); (*ii*) *Containing Putative Misannotations*, where at least one putatively misannotated protein was identified; (*iii*) *Inconclusive*, where visual inspection was insufficient to detect which protein would most likely be prone to reannotation. Groups *Containing Putative Misannotations* were further divided into two subcategories: (*i*) *Reannotated*, where all detected misannotations were revised (Additional file 1); (*ii*) *Partially reannotated*, where at least one of the putatively misannotated proteins could not be reannotated.

The error rate for *Mixed* groups was calculated as the number of *Reannotated* groups (331) divided by the sum of *Reannotated* plus *No misannotations* (442). To calculate the error rates on *Positive* and *Negative* groups, randomly chosen subsets of 169 and 291 groups, respectively, were selected and manually inspected to identify misannotated proteins. Inspection was carried out following the same criteria set for *Mixed* groups, however, proteins were only marked for reannotation but new gene models were not proposed. The Chi-square test was performed for differences among proportions, followed by the Marascuilo procedure for pairwise comparison of proportions (Stat Tools) [25]. Confidence Intervals (c.i. 95%) were calculated taking into consideration the total number of groups in each class before reannotations, 398 *Positive*, 3380 *Negative* and 541 *Mixed*.

### Search for optimized signal peptide prediction parameters

Different combinations of thresholds for signal peptide prediction parameters were tested and the total numbers of *Negative*, *Positive* and *Mixed* groups were registered at each combination. Groups containing multiple proteins per species were also considered for this analysis, and were counted as well. First, for a broad view of the entire prediction space, D-score and HMM probability values were set to start at 0.05 and were raised to 1.0 (maximum) by adding 0.05 at each iteration, while NN-Sum was tested at 1, 2, 3 and 4. Once the region with the lowest values was identified, a new round of combinations with a shorter range was run, with D-score and HMM probability values varying by 0.01, and the NN-Sum set at its optimal threshold. The combination yielding the lowest number of *Mixed* groups was chosen as the optimized set of parameters and its impact on orthologous groups was measured by determining their resulting reclassifications. Twenty-six *Negative* and *Positive* groups that changed their classification to *Mixed* after optimization were inspected in search of putative misannotated proteins.

### Functional annotation of revised genes

The ApiLoc database [26] was queried for published data on experimental localization of *Plasmodium* proteins. ApiLoc uses a structured vocabulary to describe protein subcellular localization in apicomplexan parasites. Descriptive labels were obtained for each reannotated protein and/or their respective orthologs, whenever available in ApiLoc, and were analysed in respect to which signal peptide prediction outcome would be expected for each of these proteins. The Blast2go Description Annotator (BDA) algorithm, available in the Blast2go suite (default settings: e-value cutoff of 1.0E-3, HSP length cutoff 33, nr database) [27], was used to recover the best possible description for all reannotated proteins.

### Samples, RNA extraction and cDNA synthesis

A blood sample (5 mL) was collected from a patient in the University Hospital Julio Muller in Cuiabá, MT, after the acute infection with *P. vivax* was confirmed by microscopy and written consent was given. The patient was treated according to the guidelines from the Brazilian Ministry of Health [28], and the blood sample was stored in RNALater (Invitrogen). After removal of RNALater by centrifugation the sample at 16,000 x *g* for 10 minutes, RNA extraction was carried out with TRIZOL reagent (Invitrogen) following the manufacturer's instructions. RNA was treated with RQ1 DNAse (Promega) and submitted to cDNA synthesis using the ImProm-II Reverse Transcription system (Promega) for efficient synthesis of full-length cDNAs, since amplifications were targeted to the 5' end of transcripts. To identify putative genomic DNA contamination each sample was also performed in the absence of reverse transcriptase.

### Validation of new gene models by RT-PCR

Seven reannotated proteins from *P. vivax* were selected for experimental validation of the proposed new gene models in detriment of original models. The main criterion for gene selection was that during reannotation the number of exons must have been altered, so that the different exon/intron junctions could be explored in the design of primers for RT-PCRs. For each gene, three forward (Control, Before and After) primers and one reverse primer were designed using Primer-BLAST [29] forming three pairs (Additional file 2). One of the pairs would amplify only if the original gene model were correct (Before), whereas another pair would only work in case the proposed new model was a better fit (After). The third pair was a positive control and would work on both situations. Whenever possible, the primers were designed to generate different sized fragments when amplified from genomic DNA or cDNA to avoid misinterpretation of results due to DNA contamination of RNA samples. Detailed amplification settings for each gene are in Additional file 2.

### Statistical analysis

The Chi-square test was used for assessing the statistical significance of differences between two proportions. For more than two proportions, the Chi-square was followed by the Marascuilo procedure to analyse differences between each pair of proportions. The level of significance (α) for the analyses was 0.05.

## Results

### Groups showing diverging signal peptide predictions present a higher rate of sequence misannotations

**Figure 1:**
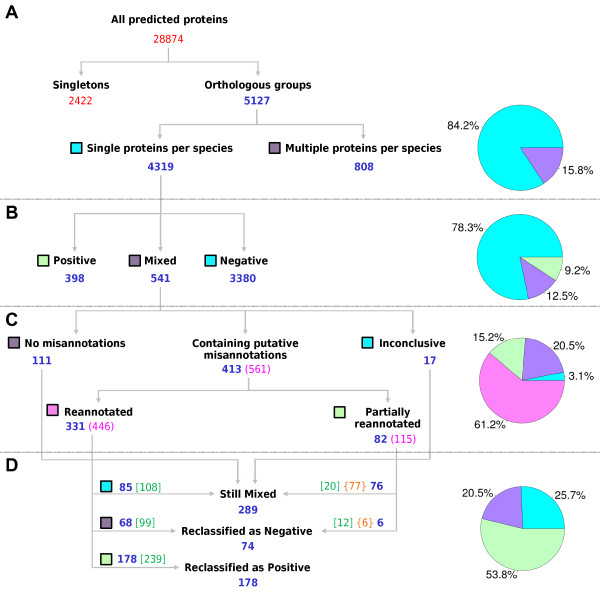
**Clustering, selection and classification of orthologous groups.** (**A**) Selection of orthologous groups. Clustering of predicted proteins from five species (*P. vivax*, *P. knowlesi*, *P. falciparum*, *P. berghei*, *P. yoelii*) according to orthologs groups defined in OrthoMCL (version 4). Numbers in blue: orthologous groups; in red: total protein numbers. (**B**) Classification of groups according to signal peptide predictions based on the status of their proteins. The numbers in blue indicate orthologous groups. (**C**) Categorization of *Mixed* groups, after visual inspection, into three categories: (*i*) *No misannotations*; (*ii*) *Containing putative misannotations*; and (*iii*) *Inconclusive*. The *Containing putative misannotations* category was divided into two subcategories: (*i*) *Reannotated*; and (*ii*) *Partially reannotated*. Numbers in blue: orthologous groups, in pink: numbers of putative misannotated proteins into each category/subcategory. (**D**) Group reclassification of signal peptide prediction after protein reannotations. Numbers in blue: orthologous groups; in green inside square brackets: reannotated proteins; in orange inside curly brackets: putatively misannotated proteins that could not be revised. To the right, graphs representing the percentages of orthologous groups in each panel, the plotted labels are indicated by square boxes matching the colors in the graphs.

**Figure 2:**
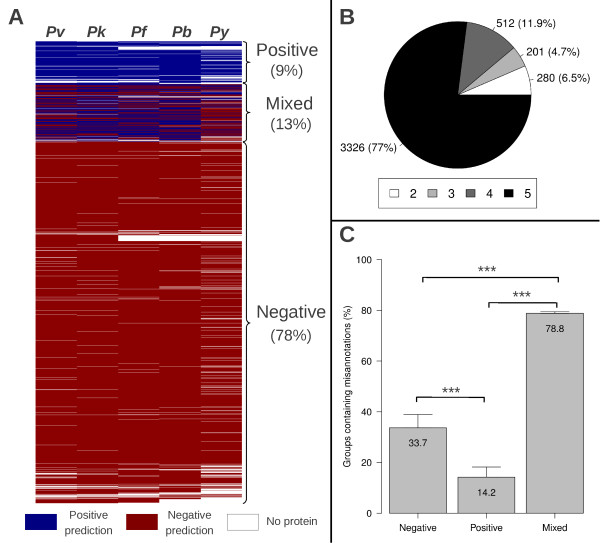
**Description of orthologous groups classified based on signal peptide prediction of their proteins.** (**A**) Distribution of 4319 groups from different *Plasmodium* species: Pv (*P. vivax*); Pk (*P. knowlesi*); Pf (*P. falciparum*); Pb (*P. berghei*); Py (*P. yoelii*). Horizontal lines represent each orthologous group. White spaces represent the lack of a protein in the species for that orthologous group. Default PlasmoDB settings were used to consider positive signal peptide predictions: NN-Sum ≥ 3 or D-Score ≥ 0.5 or HMM probability ≥ 0.5. (**B**) Distribution of number of proteins per orthologous groups, varying from 2 to 5. (**C**) Proportion of groups showing at least one misannotated protein in each of the three classes: Negative (98/291), Positive (24/169) or Mixed (330/442), error bars= 95% confidence interval. Difference among multiple proportions was measured with the Chi-square test and the Marascuilo post-hoc analysis was used for testing differences between pairs of proportions (*** p<0.0001).

Orthologous groups, defined by OrthoMCL v4.0, were used to cluster all proteins predicted in genomes of five *Plasmodium* species (*P. falciparum*, *P. vivax*, *P. knowlesi*, *P. berghei* and *P. yoelii*). There were 5,127 groups containing two or more proteins from at least one of the species studied, approximately 16% of these groups have more than one protein from the same species (Multiple proteins per species) and were excluded from further analysis (Figure 1A). The remaining 4319 groups contain from two to five proteins, with species being represented only once (Single proteins per species) (Figure 1A). Signal peptide predictions for each protein in these 4319 groups were combined with orthology information and groups were classified into the three classes: (*i*) 9.2% were *Positive* groups (all proteins have predicted signal peptides); (*ii*) 78.3% were *Negative* groups (proteins have no prediction of signal peptides); and (*iii*) 12.5% were *Mixed* groups (proteins with and without predicted signal peptides) (Figures 1B and 2A). The majority of orthologous groups have proteins from all five species (Figure 2B), reflecting their close evolutionary relationship.

Putatively misannotated proteins were identified through the visual inspection of multiple alignments of orthologs, searching for protruding or missing N-terminus that could possibly be adjusted by selecting an alternative initiation codon. Inspection of subsets from the three classes above demonstrated that the rate of groups containing at least one misannotated protein was significantly higher in *Mixed* groups (78.8%) than in *Positive* (14.2%) and *Negative* groups (33.7%) (Figure 2C).

### The majority of proposed new gene models have altered signal peptide predictions

Multiple alignments of proteins from each of the 541 Mixed groups were carefully manually inspected and sorted into three categories: (i) *No misannotations* (111 groups in Figure 1C), in which the N-terminal sequences of all proteins appear to be properly annotated (Additional file 1); (ii) *Containing putative misannotations* (413 groups), in which 561 putatively misannotated proteins were identified (Figure 1C); (iii) *Inconclusive* (17 groups in Figure 1C), for which visual inspection was insufficient to determine the annotation status of one or more proteins. The 413 groups that *Containing putative misannotations* were further divided into two subcategories: (i) *Reannotated* (331 groups in Figure 1C), in which all proteins identified as being misannotated were revised (Additional file 1); (ii) *Partially reannotated* (82 groups in Figure 1C), in which at least one putatively misannotated protein from each group could not be modified. From the 561 proteins initially selected for manual correction, 83 could not have a new gene model proposed (orange numbers in curly brackets in Figure 1D). For most of them it was because of missing sequence information in the upstream flanking region of the gene due to incomplete genome assembly and in one case there was a frame shift in the middle of an exon, interpreted as a possible sequencing error that prevented the reannotation. Some of the groups *Partially reannotated* presented some proteins that were reannotated (in addition to those that were only marked), thus, six of these groups were reclassified as negative even though groups were not fully reannotated (Figure 1D). A total of 478 proteins had their gene models revised and their amino acid sequences reannotated (green numbers in square brackets in Figure 1D).

**Figure 3:**
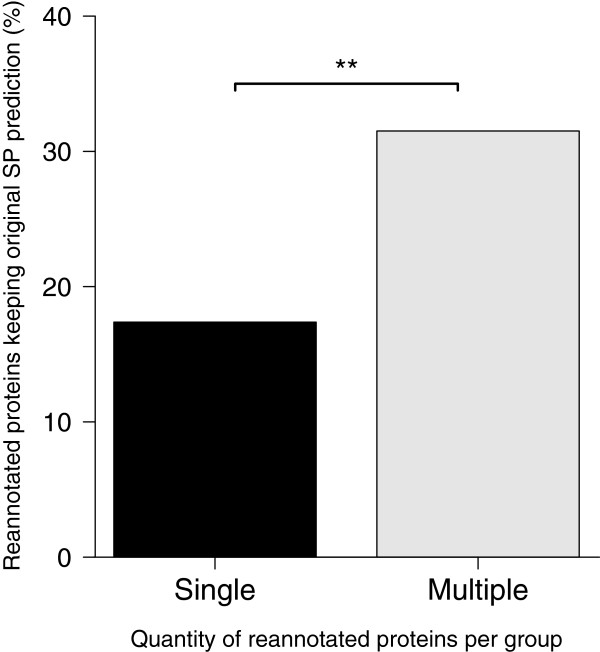
**Proportion of reannotated proteins keeping original signal peptide predictions in groups with single or multiple revised proteins.** All reannotated proteins were divided in two categories: Single – groups with only one reannotated protein (N=259) and Multiple - groups with at least two reannotated proteins (N=219). The numbers of proteins with unchanged signal peptide prediction in each category were 45 (Single) and 69 (Multiple). The Chi-square test was used to calculate statistical significance of the differences between proportions (** p<0.001)

A total of 364 (~75%) proteins had their signal peptide predictions altered, with changes to positive signal peptide predictions being the most common alteration observed in reannotated proteins (Table 1). A total of 114 (~24%) proteins, albeit having had their sequences modified through reannotation, kept their original signal peptide predictions (Table 1). However, these proteins were more frequently found in groups that had multiple proteins reannotated (Figure 3) and were probably selected for reannotation only as by-products of the reannotation process.

**Table 1:** **Signal peptide predictions of proteins from *Plasmodium *species after reannotations**

	**Signal peptide prediction of reannotated proteins**				**Total proteins with positive signal peptide predictions**		
**Species**	**Became Negative**	**Became Positive**	**Same Prediction**	**TOTAL**	**Before**	**After**	**Af – Bf (%)**
*P. vivax*	16	105	37	158	822	911	89 (10.8%)
*P. knowlesi*	15	29	21	65	840	854	14 (1.7%)
*P. falciparum*	5	15	8	28	1057	1067	10 (0.9%)
*P. berghei*	8	5	6	19	807	804	-3 (-0.4%)
*P. yoelii*	41	125	42	208	983	1067	84 (8.5%)
TOTAL	85	279	114	478	4509	4703	194 (4.3%)

*Plasmodium yoelii* followed by *P. vivax* were the two species presenting highest numbers of reannotated proteins, 208 and 158, respectively (Table 1). This might reflect the overall annotation status of genomes from these species when compared to others more extensively studied such as *P. berghei* and *P. falciparum*. Apart from *P. berghei*, all the other four species showed, as a net result, an increased number of positively predicted proteins, especially *P. vivax* and *P. yoelii*, which had over 80 proteins added to their previous counts of proteins featuring signal peptides. Proportionately, *P. vivax* was the species that showed the highest impact from reannotations in its final count of positively predicted proteins (Table 1). The complete list of reannotated proteins, with their new proposed sequences is available (Additional file 3).

### Reannotations alter the classification of groups

**Figure 4:**
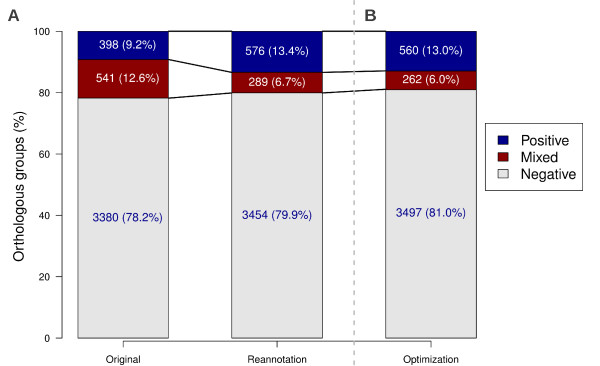
**Protein reannotations and optimization of signal peptide prediction parameters influencing the classes of orthologous groups.** (**A**) Reclassification of groups based on signal peptide prediction due to reannotation of proteins from *Mixed* groups. Default PlasmoDB settings were used to consider positive signal peptide predictions: NN-Sum ≥ 3 or D-Score ≥ 0.5 or HMM probability ≥ 0.5. (**B**) Further reclassification of groups due to optimization of signal peptide threshold prediction parameters. The lowest number of Mixed groups was achieved by resetting thresholds to: NN-Sum = 4; D-Score = 0.48 and HMM probability = 0.9.

Before proteins were reannotated, *Mixed* groups were more numerous than *Positive* groups. However, as previously demonstrated, the reannotations resulted in changed prediction status for most proteins, consequently impacting on the number of *Mixed* groups, as 74 were reclassified as *Negative* and 178 were reclassified as *Positive* groups (Figure 1C and D). The original 541 *Mixed* groups were reduced to 289 and proportionately their representation dropped to only 6.7% of groups, whereas *Positive* and *Negative* groups both increased their representation to 13.4% and 79.9%, respectively (Figure 4A). The inversion observed between the percentage of *Positive* and *Mixed* groups suggests that the divergence of signal peptide predictions among orthologous proteins is indeed a rare event, especially for proteins from closely related species (belonging to the same genus).

### Transcripts expression supports the new gene models

**Figure 5:**
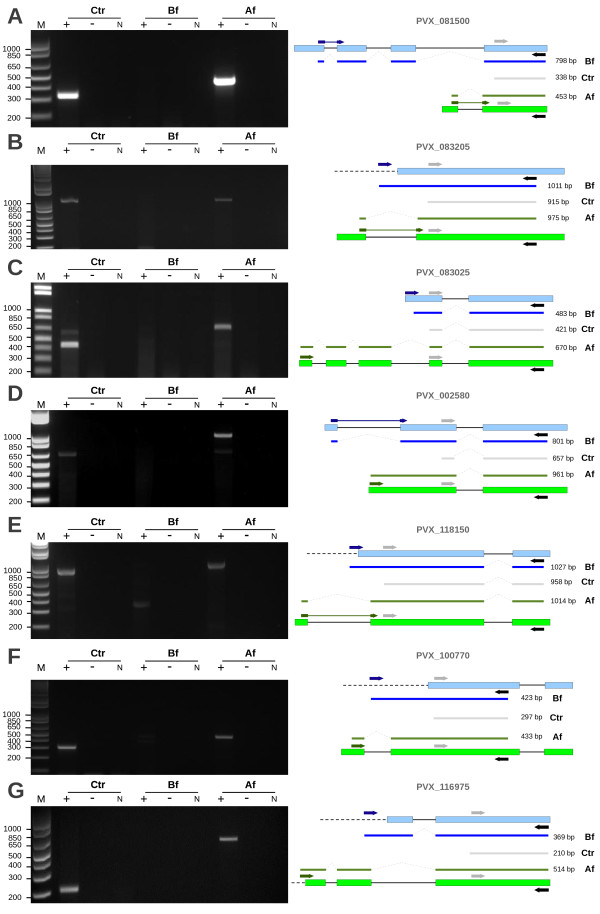
**Experimental validation of proposed new gene models.** Left panels show the amplification of *Plasmodium vivax* cDNA isolated from an infected patient. Right panels show schematic representations of the original gene model (light blue boxes) and the new model (light green boxes). PCRs were done using Control (light grey arrow), Before (dark blue arrow) and After (dark green arrow) forward primers with the same Reverse (black arrow) primer in the presence (+) or absence (-) of reverse transcriptase. The resulting amplicons (Before – blue line; Control – grey line; After – green line), with their respective molecular sizes, are shown in the middle of right panels for genes encoding for proteins (description according to BDA results): [PlasmoDB:PVX_081500] adenyl cyclase associated protein (**A**), [PlasmoDB:PVX_083205] protein transport protein Sec61 alpha subunit (**B**), [PlasmoDB:PVX_083025] sporozoite microneme protein (**C**), [PlasmoDB:PVX_002580] pseudouridine synthetase (**D**), [PlasmoDB:PVX_118150] glutamine cyclotransferase (**E**), [PlasmoDB:PVX_100770] conserved hypothetical protein (**F**) and [PlasmoDB:PVX_116975] conserved hypothetical protein (**G**). Dashed linear lines in gene models represent the 5' UTR of mRNAs. N – negative PCR control (without DNA); M - Molecular marker (1Kb Plus, Invitrogen). The schematic representations of the gene models were not in scale.

The numbers of exons as well as exon/intron junctions in their N-terminal region where signal peptides are usually found have changed for several reannotated proteins. Seven genes were selected for assessing the verisimilitude of their proposed reannotations. The primers designed to confirm the new gene models were able to amplify products with the expected sizes for all of them (Figure 5). Both negative (in the absence of reverse transcriptase) and positive (using control primers) controls worked properly, corroborating the new gene models proposed. Three of the validated genes became positive after reannotations [PlasmoDB:PVX_002580, PlasmoDB:PVX_083025, PlasmoDB:PVX_100770], three became negative [PlasmoDB:PVX_081500, PlasmoDB:PVX_116975, PlasmoDB:PVX_118150] and one [PlasmoDB:PVX_083205] kept its original negative prediction (Additional file 3).

### Reannotated proteins have functional annotation support

Since the biological roles of proteins are ultimately influenced by the presence or absence of signal peptides, the functional annotations of reannotated proteins were analysed. First, experimental evidence available for reannotated proteins or their orthologs were searched in the ApiLoc database. Only 8 out of 478 reannotated proteins have already had their localizations directly confirmed by experimental approaches, and all show a positive SP prediction, with 6 having turned positive only after reannotation (Table 2). Subcellular localizations that rely on the presence of a signal peptide were described for 7 of these proteins, with one of them [PlasmoDB:PF14_0517] localizing to the food vacuole but also to the cytosol. The only reannotated protein that had a SP prediction conflicting with its experimentally verified localization was [PlasmoDB:PFB0400w] (6-cysteine protein). Moreover, although this protein had its sequence reannotated, its SP prediction was already positive, which means that the disagreement was not due to the reannotation.

**Table 2:** Reannotated proteins with direct experimental validation of subcellular localization

**PlasmoDB Gene ID**	**Species**	**Ortholog group**	**Signal peptide prediction**		ApiLoc*	
			**Before**	**After**	**Localization**	**Reference**
PF14_0517	*Pf*	OG4_10729	Negative	Positive	Cytosol and food vacuole during trophozoite	[60]
PFB0400w	*Pf*	OG4_42799	Positive	Positive	Cytoplasm during gametocyte stage v	[46]
PVX_090075	*Pv*	OG4_54213	Negative	Positive	Rhoptry during schizont	[45]
PY03011	*Py*	OG4_48314	Negative	Positive	Apical and basal and not nucleus during salivary gland sporozoite	[61]
PY00454	*Py*	OG4_21677	Positive	Positive	Microneme during sporozoite	[62]
PY00819	*Py*	OG4_10672	Negative	Positive	Apicoplast during hepatocyte schizont and salivary gland sporozoite	[63]
PY07092	*Py*	OG4_47550	Negative	Positive	Apical and not surface during salivary gland sporozoite	[61]
PY04986	*Py*	OG4_25099	Negative	Positive	Apical during oocyst, sporozoite	[64]

*Recovery of experimental data from the literature, available in the ApiLoc database, describing the localization and timing of expression for reannotated proteins. Species: *Pf Plasmodium falciparum*, *Pv Plasmodium vivax*, *Py Plasmodium yoelii.*

In addition, there are 57 reannotated proteins whose orthologs have experimental evidence of cellular localization (Additional file 4). For 40 of these proteins, final SP predictions were positive and, impressively, 39 (98%) of them have orthologs whose localizations concur with the presence of a signal peptide. [PlasmoDB:PVX_117660] (serine hydroxymethyltransferase) is the only reannotated protein whose positive SP prediction clearly conflicts with the experimental validation of its ortholog [PlasmoDB:PF14_0534], which does not have a signal peptide and localizes to the mitochondrion. Of the 17 reannotated proteins with negative SP predictions and experimentally validated localization of orthologs, only 8 show experimental data agreeing with an absent signal peptide. However, 5 out of the 9 remaining proteins already had negative predictions before reannotation. Also, SP predictions are negative for the orthologs of 8 out of these 9 proteins as well, despite their experimental localizations suggesting the presence of signal peptides (Additional file 4).

Gene product descriptions were also considered as hints on putative biological functions. However, approximately 60% of reannotated proteins are described as unknown or hypothetical according to PlasmoDB (v7.1). Thus, protein description was complemented by running the Blast2go Description Annotator (BDA) and hypothetical/unknown proteins were reduced to approximately 35% only (Additional file 3). After that, the focus of the functional analysis on *P. vivax* proteins was in the relevancy of proposed reannotations and how they may contribute in efforts to control malaria. In addition to the above mentioned experimental evidences involving *P. vivax* proteins, the level of agreement between reannotation outcome and product description for all reannotated proteins in this species was analysed. Out of 158 reannotated proteins, 53 are described as hypothetical or unknown and could not be analysed. Out of the remaining 105 proteins, product descriptions accorded with signal peptide predictions (after reannotation) for 78, disagreed for 15 and were considered inconclusive (insufficient descriptive information) for 12 proteins (Additional file 3).

Among the proteins that turned to a positive SP prediction there are 4 tRNA-synthetases, 1 tRNA-amidotransferase (GATase), 2 translation initiation factors, 2 DNA gyrase subunits and 1 ferredoxin (Table 3). The first seven are involved in transcription/translation of proteins, the gyrase subunits participate in DNA replication and the exact function of the ferredoxin remains unkonwn. The *P. falciparum* orthologs for these 10 proteins all have signal peptides and localize to the apicoplast. After the proposed reannotations, the *P. vivax* proteins are in perfect parallelism to their *P. falciparum* orthologs, including the orthology affiliations of the negatively predicted counterparts for the tRNA-synthetases and gyrases.

**Table 3:** ***Plasmodium falciparum *orthologs supporting signal peptide predictions of *Plasmodium vivax *reannotated proteins**

		***P. falciparum *ortholog**			***P. vivax *ortholog**	
**Protein description**	**Ortholog group**	**PlasmoDB Gene ID**	**SP prediction**	**Predicted localization**	**PlasmoDB Gene ID**	SP prediction*
Proline tRNA synthetase	OG4_11662	PFI1240c	**+**	Apicoplast	PVX_099680 ®	**+**
	OG4_10599	PFL0670c	**-**	Cytosol	PVX_123380	**-**
Asparagine tRNA synthetase	OG4_44547	PFE0475w	**+**	Apicoplast	PVX_098040 ®	**+**
	OG4_10251	PFB0525w	**-**	Cytosol	PVX_002940	**-**
Methionine tRNA synthetase	OG4_47490	PF10_0053	**+**	Apicoplast	PVX_094445 ®	**+**
	OG4_10161	PF10_0340	**-**	Cytosol	PVX_110980	**-**
Leucine tRNA synthetase	OG4_11105	PF08_0011	**+**	Apicoplast	PVX_088945 ®	**+**
	OG4_10828	PFF1095w	**-**	Cytosol	PVX_114255	**-**
Glutamate tRNA amidotransferase subunit A	OG4_10803	PFD0780w	**+**	Apicoplast	PVX_089895 ®	**+**
Initiation Factor-1	OG4_12019	MAL8P1.27	**+**	Apicoplast	PVX_089095 ®	**+**
Initiation Factor-3	OG4_14117	PF14_0658	**+**	Apicoplast	PVX_117015 ®	**+**
DNA gyrase subunit A	OG4_12828	PFL1120c	**+**	Apicoplast	PVX_123795 ®	**+**
	OG4_10491	PF14_0316	**-**	Nucleus	PVX_084855	**-**
DNA gyrase subunit B	OG4_12159	PFL1915w	**+**	Apicoplast	PVX_100925 ®	**+**
	OG4_10746	PF10_0412	**-**	Nucleus	PVX_111340	**-**
Ferredoxin	OG4_14702	MAL13P1.95	**+**	Apicoplast	PVX_122725 ®	**+**

® Reannotated proteins.

* Signal peptide prediction result for the new sequence if the protein has been reannotated.

### Mixed groups can be further reduced by optimization of signal peptide prediction parameters

**Figure 6:**
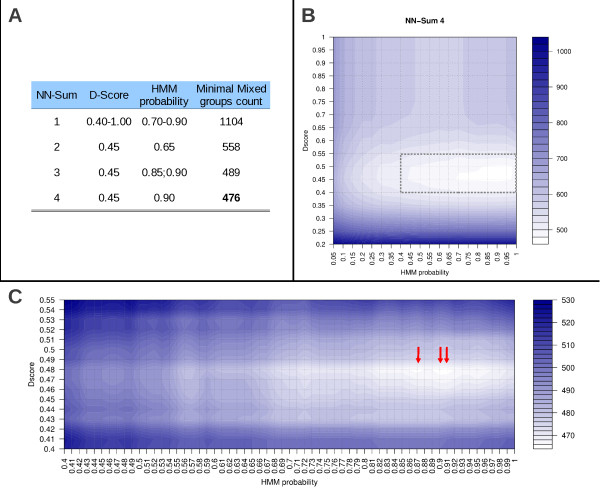
**Optimization of signal peptide threshold prediction parameters searching for the lowest number of *Mixed *groups.** Several combinations of the three parameters used for signal peptide prediction in PlasmoDB were tested in search of the optimal setting. (**A**) The first analysis was all possible values of NN-Sum combining with D-Score and HMM probability ranging from 0.05 to 1.0 (intervals of 0.05). (**B**) Graphic representation of this combination is showed for the optimal threshold of NN-Sum = 4. The area registering the lower numbers of *Mixed* groups (dotted rectangle) for a refined search using intervals of 0.01 units (**C**). The lowest number of Mixed groups (465) was achieved by resetting thresholds to: NN-Sum = 4; D-Score = 0.48 and HMM probability = 0.87; 0.90; 0.9 (indicated by the red arrows).

Although reannotations resulted in a significant reduction of *Mixed* groups, there are other interventions that could translate into further reclassification of *Mixed* groups. In line with the proposed hypothesis, erroneous predictions of signal peptides could create artificial *Mixed* groups or even conceal genuine *Mixed* groups, therefore the impact of changing prediction parameters on the classification of groups was tested. Optimization was carried out only once, after reannotation of proteins, and optimal threshold values were defined as the combination resulting in the lowest number of *Mixed* groups. Initially, the best NN-Sum threshold was identified at 4, which in combination to D-score at 0.45 and HMM probability at 0.90 defined 476 *Mixed* groups (including groups with multiple proteins per species) (Figure 6A). These results were further refined within a shorter range (Figure 6C), previously delimited (dotted square in Figure 6B), where 465 *Mixed groups* were identified using the best threshold combination: NN-Sum=4; D-Score=0.48; HMM probability=0.87, 0.90 or 0.91 (Figure 6C). HMM probability of 0.90 was chosen as the optimal threshold for further analysis as it was the most representative of the three HMM probabilities obtained. Applying these settings to the 4319 groups (Figure 1B) further reduced the number of *Mixed* groups from 289 (after reannotation) to 262 (Figure 4B). A total of 61 original *Mixed* groups were reclassified as Negative (50) or Positive (11) (Table 4 and Additional file 5) due to optimization, however, optimization also led to the reclassification of 34 Positive (26) and Negative (8) groups as Mixed (Table 4 and Additional file 5), explaining the small difference between default and new settings. Among the 34 groups reclassified as Mixed, 8 were actually reverting to their original classification after they were reclassified as Positive after reannotations. In addition, the other 26 groups that were also reclassified as Mixed were submitted to visual inspection and the need for reannotation was revealed in 13 (50%) of them (Additional file 5), indicating that optimization was indeed beneficial as more groups, previously overlooked for not being classified as Mixed, were considered for reannotation. Interestingly, optimization of signal peptide prediction parameters caused the reclassification of *Mixed* groups (61/289, 21.1%) in a greater proportion than *Positive* (26/576 4.5%) and *Negative* groups (8/3454 0.002%), indicating that parameter optimization preferentially acts upon those groups in which spurious divergences between orthologs are more likely to be observed.

**Table 4:** Number of orthologous groups in signal peptide group classes after optimization of signal peptide prediction parameters

**Group Classes**	Before optimization*	After optimization**			
		**Negative**	**Positive**	**Mixed**	**Total**
Negative	3454	-	0	8	3496
Positive	576	0	-	26	561
Mixed	289	50	11	-	262
Total	4319	50	11	34	4319

*NN-Sum=3; Dscore=0.50; HMM probability=0.50.

**NN-Sum=4; Dscore=0.48; HMM probability=0.90.

### Signal peptide patterns mirroring the phylogeny of *Plasmodium* are more common in groups consistently classified as Mixed

**Figure 7:**
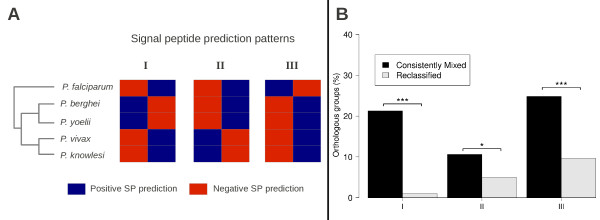
**Signal peptide prediction patterns among *Plasmodium *species.** (**A**) Three distinct patterns of signal peptide predictions were compared to a schematic phylogenetic tree (based on mitochondrial genes [59]) of five *Plasmodium* species to represent a likely evolutionary support. Pattern I: *P. berguei* and *P. yoelii*; Pattern II: *P. vivax* and *P. knowlesi*; Pattern III: *P. falciparum*. (**B**) The proportions of these three patterns were compared between groups that were originally Mixed but were reclassified because of either reannotations or optimization of signal peptide prediction parameters (N=301) and groups that have retained their classification as Mixed group despite inspections, reannotations and optimization (N=141). The Chi-square test was used to calculate statistical significance of the differences between proportions (* p<0.05, *** p<0.0001).

Reannotations and/or optimization of prediction parameters may change the original classification of groups (Figure 4). However, 228 groups have retained their mixed classification throughout (Additional file 5). Among these 228 groups, there are 87 (70 groups *Partially reannotated* and the 17 *Inconclusive* groups) that could still be reclassified as a result of future reannotations and were excluded from this analysis. The remaining 141 groups are still classified as *Mixed* (Additional file 5) despite having already undergone inspection, reannotation or parameter optimization. Therefore, according to the proposed hypothesis, it is likely that true biological diversity might be found among these 141 groups, as a consequence of orthologs genuinely evolving to have divergent signal peptide states. There are three signal peptide prediction patterns that mirror the phylogeny of the *Plasmodium* genus, with divergence being restricted to *(i) P. falciparum*, *(ii)* Rodent parasites (*P. berguei* and *P. yoelii*), or *(iii) P. vivax* and *P. knowlesi* (Figure 7A). Interestingly, these 141 groups show higher proportions of these patterns when compared to 301 orthologous groups that were originally *Mixed* but have been reclassified either due to reannotations or optimization (Figure 7B). This correspondence between signal peptide predictions and phylogeny supports the notion of biological novelties within these groups.

## Discussion

The approach intended in this work shifts the perception of signal peptide data as an exclusive property of individual proteins to a perspective where it also becomes a descriptive characteristic of orthologous groups of proteins, with groups being classified into three distinct categories: Positive, Negative or Mixed. It is important to note that paralogs, in general, evolve diverging functions more rapidly and more often than orthologs [12,30]. Therefore, the expected conservation of signal peptide predictions among orthologs does not necessarily hold true for paralogs, as demonstrated for *P. vivax* VIR protein family [31]. Even though OrthoMCL will only cluster recent paralogs [20], groups containing multiple proteins per species were excluded from the reannotation analyses. This was done because, generally, paralogs are not as conserved as orthologs in function and, consequently, in signal peptide state, according to the ortholog conjecture [32]. The ortholog conjecture is the paradigm behind the widespread use of orthology in comparative biology, however, it has always been a rather theoretical proposition, and only recently it was put to the test. Some studies have contested its validity, especially for the direct link made between function and sequence similarity. In one particular study, paralogs were shown to be better predictors of function than orthologs [33]. On the other hand, the ortholog conjecture has been reaffirmed by other studies that showed significantly more conservation of protein structure and expression profiles among orthologs than paralogs [32]. These recent studies clearly signal that the debate is still open. The conservation of signal peptides has not been addressed directly until now.

The strategy was applied to the predicted protein sets of five *Plasmodium* species and found that an expressive number of proteins showed diverging signal peptide predictions when compared to their orthologs. The rate of *Mixed* groups observed was higher than expected, considering the rarity of divergence and the close evolutionary proximity of the species studied (same genus). Therefore, a few probable explanations were considered: (*i*) Misannotated proteins, particularly their N-terminal end; (*ii*) Errors or shortcomings in the predicting programs; and (*iii*) Biological diversity due to divergence in the course of evolution, which constituted the real Mixed groups.

Misannotated sequences were the most likely source of diverging signal peptide predictions. It is known that definition of initial methionine is the most challenging tasks for gene annotating algorithms, particularly for eukaryotes, which means that annotation of the N-terminal end of proteins, exactly where most signal peptides are found, is intrinsically less accurate [34]. The majority of *Mixed* groups had at least one protein that needed N-terminal sequence reannotation. Comparing *Positive* and *Negative* groups, the rate of misannotated proteins in *Mixed* groups is much higher, signaling that the combinatory strategy was efficient for enrichment of misannotated sequences, a desirable trait in a quality control mechanism for sequence accuracy in genomic scale.

Most protein reannotations resulted in altered signal peptide predictions, which in turn were converted into the reclassification of orthologous groups. The new distribution of *Positive*, *Mixed* and *Negative* groups demonstrates that having orthologs drastically diverging in their putative subcellular targeting is far less usual than previously shown, and this erroneous interpretation was mostly due to sequence misannotation. The observed reduction of *Mixed* groups from 541 to 289 due to reannotation is, indeed, a conservative estimate as additional reannotations are still a possibility for the 17 Inconclusive groups and the 82 groups *Partially reannotated*, in which there are proteins that could not be reannotated at this moment. Therefore, the eventual correction of these groups could result in an even lower rate of *Mixed* groups.

The main reason preventing the reannotation of proteins from groups *Partially reannotated* was the truncation of the upstream flanking region. This is directly related to the assembly states of genomes and explains why *P. yoelii* genes were most affected. According to PlasmoDB (v7.1), among the studied species, *P. yoelii* has the genome with the highest count of unassigned contigs (5687), followed by *P. vivax* (2770). Another reflection of the assembly state of *P. yoelii* genome is made clear in Figure 2A, in which proteins from this species seem to be missing from several orthologous groups. Improvements in the genome assembly would likely result in the identification of these missing orthologs by gene prediction algorithms.

Sequence misannotations are more likely to generate negatively predicted proteins. Since signal peptides are defined by typical structural constrains [35], the probability that any randomly chosen amino acid stretch (≥ 40 amino acids), coded by a genomic sequence and having a methionine in the first position, will hold a signal peptide is lower than otherwise (data not shown). Therefore, proteins with wrongly assigned initial methionine tend to show negative signal peptide predictions. Thus, while most proteins without signal peptide will preserve their signal peptide predictions even if misannotated, most proteins with signal peptide will have their predictions inverted when misannotated. This uneven effect explains why the rate of misannotations is higher in *Negative* than in *Positive* groups and why most suggested reannotations have resulted in proteins turning from negative to positive predictions. The underlying message is that, as a rule, this particular reannotation strategy tends to increase the set of proteins predicted to have signal peptides, as demonstrated for four out of the five species studied, and this biased enrichment of positive proteins may be beneficial in the search for new vaccine targets.

In an effort to understand the persistent classification of some groups as *Mixed*, signal peptide prediction itself was also investigated as a source of divergence among orthologs. When combining orthology and signal peptide information, the default settings applied by PlasmoDB for signal peptide prediction were used, however, there were concerns on how well adjusted were these settings, and whether was there room for improvement. With the intention of avoiding or, at least, reducing the number of false *Mixed* groups created by faulty predictions, it was reasoned that optimal prediction conditions would be found when predictions among orthologs reached their highest level of agreement, minimizing the number of *Mixed* groups. Optimization was carried out only once, after reannotations were incorporated to the database, when ideally, parameter optimization and sequence reannotations should work to complement each other in an iterative process, with new reannotations being incorporated at each round and optimal conditions being recalculated afterwards. Therefore, the new thresholds suggested here should be considered with caution because there are still many factors that could cause further alterations (new reannotations, incorporation of new genes, changes in orthology), and should not be taken as definitive values.

Also, optimization by itself does not correct intrinsic software limitations such as a biased training dataset. Although SignalP is a robust application and has been widely employed, its eukaryotic training dataset is dominated by mammalian sequences [36] and it is possible that signal peptides from *Plasmodium* proteins are somewhat different from those of mammals. This difference alone could be responsible for overestimation of the divergence. Nonetheless, the results offer a refreshing view on how to improve signal peptide predictions within clusters of species without having to implement major changes in existing prediction softwares, and it could also contribute to the development of predictors as *Mixed* groups may help identify which sequences are beyond current detection limits and should, therefore, be incorporated in future training data sets.

Pre-calculated orthology clustering was chosen over an independent assessment because this information is readily available for download from PlasmoDB reflecting the resources available to the malaria community. For the same reason, PlasmoDB’s SignalP prediction settings were used instead of settings from SignalP standalone version. Also, by using pre-calculated clustering the strategy became less computationally demanding. Independent clustering could have an impact in reannotations, as groups could have been added or lost, but the major results and the overall conclusions would not change. Last, considering the evolutionary proximity of the studied species and the high conservation observed among orthologs in most groups, clustering would not vary much from that obtained from OrthoMCL.

Biological features of *Plasmodium* could also justify difficulties in signal peptide prediction. Some *Plasmodium* secretory proteins use ‘unconventional protein secretion’ which collectively describe several kinds of unusual trafficking pathways that lead to the exposure of proteins on cell surfaces or to their release into the extracellular space [37,38]. This includes Golgi-independent trafficking of integral membrane proteins [39] and other variations of transport modes within the classical secretory pathway [37,38]. In these cases, typical signal peptides are not present, and many known secreted proteins of *Plasmodium* are included in this category, for example, RESA, GBP-130, Pf41-2, PfHPRT, FIRA, among others [40]. However, even for these proteins the expected conservation of signal peptide prediction state is applicable. If a given protein is trafficked via an alternative route and features a negative signal peptide prediction, the same result is to be expected from its orthologs, as they would also be subjected to the same biological processing.

*Plasmodium* has a very complex life cycle with multiple invasion steps mediated by highly specialized apical organelles (rhoptries, micronemes and dense granules), and targeting to these organelles is signal peptide dependent [41]. Once invaded, red blood cells (RBCs) are remodeled by *Plasmodium* in a process that involves the export of several parasite proteins to the cytoplasm and membrane surface of RBCs [42]. And again, signal peptides are required for allowing entrance into the ER and subsequent targeting to the parasitophorous vacuole (PV) lumen, the default secretory pathway for *P. falciparum* proteins [43]. Biological diversity within the *Plasmodium* genus is also a possible explanation for *Mixed* orthologous groups, and the implications of divergent orthologs are rather interesting, as they are likely to be involved in processes that are unique to a few or even one organism. In *Plasmodium*, these genes could mediate or interfere with any of the several singular phenomena that set species apart, such as sequestration in *P. falciparum*, host cell invasion preferences of merozoite, variability in the maturation or morphology of gametocytes or the formation of latent stages in *P. vivax*[44]. Identification of such instances, where interspecific diversity could be occurring, is of utmost relevance to malariology. However, unequivocal demonstration of biological divergence, in terms of protein localization, demands experimental procedures (i.e.: fluorescent protein tagging, immunohistochemistry with specific antibodies), which are beyond the scope of this work. Nonetheless, the likelihood of finding true biological diversity was narrowed to a subset of 141 groups that have kept their mixed classification despite efforts of reannotation and optimization. Interestingly, signal peptide prediction patterns that concur with the phylogeny of *Plasmodium* species were significantly over represented in these groups, which argues in favor of biological novelties as the observed divergences could then be attributed to the particular evolutionary history of each species. The proteins from these groups in particular warrant further studies to confirm or reject their link to biological phenomena restricted to subsets of *Plasmodium* species.

The reannotations being proposed redefine the sets of proteins that are targeted to the ER of *Plasmodium* organisms and are highly relevant, since protein trafficking is crucial for the successful development of these organisms within their hosts. Therefore, direct as well as indirect experimental evidences were important to support reannotations. Although validation of new gene models through RT-PCR does not allow proper identification of initial methionine, it clearly demonstrated that the new gene models are a good fit to the mRNAs being expressed by parasites, whereas original gene models were not. Only some reannotated proteins were prone to RT-PCR validation as a difference in the number of exons or a modification of exon/intron boundaries between original and new gene models are required. Apart from this prerequisite, targets for validations were chosen so both inversion and maintenance of signal peptide prediction cases were covered.

Available evidences of protein localization and their correlation to signal peptide predictions for the new protein sequences were also analysed. Although only eight have been experimentally validated, most of their localizations are in accordance to their newfound signal peptide predictions. In fact, one of them [PlasmoDB:PVX_090075], a protein localized in the rhoptries, has been characterized as a promising vaccine candidate capable of eliciting a humoral immune response and the proliferation of lymphocytes from human patients [45]. The only conflicting protein is a male-specific protein [PlasmoDB:PFB0400w] said to be cytoplasmatic according to immunofluorescence assays, however its patchy and diffuse pattern coupled to secretory signal sequence, also identified in the manuscript, suggested that the protein may be located in cytoplasmic vesicles instead [46]. Even when experimental evidence from orthologs were considered, signal peptide prediction of a given protein and the localization of its orthologs were highly agreeable. Out of 40 proteins with positive predictions, only one [PlasmoDB:PVX_117660] shows a signal peptide prediction incompatible with its ortholog’s localization. However, this protein shows a positive prediction, even before reannotation and it is the only protein in its orthologous group with a signal peptide, thus it remains to be experimentally demonstrated whether this *P. vivax* protein is indeed different from its orthologs. Among the negatively predicted proteins, concordance to orthologous localizations was lower, however, most signal peptide predictions from the orthologs themselves conflicted with their localizations. A possible explanation for contradiction between negatively predicted proteins and subcellular localization might be alternative sorting routes independent of signal peptides as discussed before.

Another challenge for signal peptide prediction in *Plasmodium* species is the presence of a unique organelle from apicomplexa resulting from secondary endosymbiosis, the apicoplast. This organelle is an active site of protein transcription/translation and DNA replication [47,48]. Pharmacological and genetic perturbation of the apicoplast led to parasite death [49,50] and it was recently described that the essential function of the apicoplast is biosynthesis of an isoprenoid precursor during the blood-stage growth. Its essentiality for the parasite survival and the absence of a metabolic counterpart in human host make the apicoplast proteins promising targets for anti-malarial drug development [51]. As only a few proteins (~50 mostly housekeeping genes) are encoded in the organellar genome [52], most of apicoplast housed proteins (~500 proteins), coded in the nuclear genome, must be transported to the apicoplast, via a mechanism mediated by a bipartite sorting element formed by a signal peptide followed by a transit peptide [53]. Several of the reannotated proteins that became positively predicted have orthologs that are apicoplast-targeted, demonstrating how these reannotation efforts may assist in the quest for new anti-malarial drugs.

The search for new intervention targets for disease control is increasingly dependent on computational approaches that query and filter vast amounts of biological data, which makes annotation accuracy a priority since imprecise inputs will return low quality results. Signal peptides, for example, are extensively employed as a filter in reverse vaccinology strategies [54], as targets for humoral response are usually secreted or surface attached proteins, and misinformation on protein N-terminal sequences would certainly prevent correct identification of putative targets. Most of the major *Plasmodium* vaccine candidates (i.e.: AMA-1, Pfs230, CS, PvDBP) [55] are proteins that have predicted signal peptides, demonstrating how important this feature can be in the discovery of new vaccine targets. Also, information on signal peptides can be incorporated in the process of selecting drug targets when it is known or expected that the metabolic process to suffer intervention takes place in membrane bound organelles or cellular compartments. Once more, *Plasmodium* stands as a good example, as it has already been demonstrated that the food vacuole [56] and the apicoplast [49] are susceptible to anti-malarial compounds, and protein targeting to both these organelles is signal peptide dependent [57]. Apicoplast targeting was one of the filtering criteria for identifying attractive drug targets in *Plasmodium falciparum* in a study that used a comprehensive *in silico* approach [58].

## Conclusions

The combinatorial strategy presented here proved to be a powerful tool for identification of misannotated N-terminal sequences, and allowed the redefinition of the list of proteins destined for ER targeting in five *Plasmodium* species. It might have an important impact in the available data for genome-wide searching of potential vaccine and drug targets and proteins involved in host/parasite interactions, particularly for *P. vivax*. Most of the proposed reannotations are already available in PlasmoDB as user comments, and the remaining set will be uploaded shortly.

This study suggests that misannotated proteins are frequently found in genome databases, reflecting limitations and shortcomings of the gene prediction algorithms used in genome annotations. Therefore, new strategies incorporating additional information, such as signal peptide prediction to these algorithms may improve the annotation process. Moreover, despite the analyses were restrained to *Plasmodium* species at the moment, this strategy can be readily applied to the predicted proteins of any cluster of species in order to assist in efforts to curate protein sequence information.

## Abbreviations

NN: Neural network; HMM: Hidden Markov Model; CDS: Coding sequence.

## Competing interests

The authors declare that they have no competing interests.

## Authors’ contribution

AMN performed all data analyses. AMN, RSR and AMR work on scripts for data integration. DAM and SSR participated in the experimental validation of new gene models. CJFF coordinated the field work. LHC, CFAB, AMN conceived and designed the study. CFAB coordinated the study. CFAB, AMN and AMR wrote the manuscript. All authors read and approved the final manuscript.

## Supplementary Material

Additional file 1 **Examples of N-terminal alignments of inspected Mixed groups.** In the upper panel, three Mixed groups (OG4_10598, OG4_10633 and OG4_47034) placed in the category of No misannotations after visual inspection. Signal peptide predictions positive (+) or negative (-) are shown to the left of gene identifiers, demonstrating the Mixed nature of these groups. A total of 111 groups belong to this category. In the lower panel, two Mixed groups (OG4_54958 and OG4_54960) in which putative misannotated proteins were identified after visual inspection. Proteins in these groups were reannotated and a comparison of alignments before and after reannotations is shown with the respective signal peptide predictions to the left of gene identifiers. A total of 331 groups belong to this category (Reannotated). Reannotated *P. vivax* genes that were submitted to RT-PCR validation of new gene models are indicated by asterisks. Click here for file

Additional file 2 **Description of PCR conditions used to validate new gene models.** Sequences of primers, amplicon sizes, annealing temperatures and number of cycles used in amplifications of seven new gene models are showed. For each gene model were used three forward primers (control, before and after) and the same reverse primer. Click here for file

Additional file 3 **List of all reannotated proteins.** Reannotated proteins identified by their Gene ID from each species are listed including their orthologous group number, signal peptide prediction before and after annotation, description of putative gene product and new sequence proposed. Species: Pb – *Plasmodium berghei*, Pf – *Plasmodium falciparum*, Pk – *Plasmodium knowlesi*, Pv – *Plasmodium vivax*, and Py – *Plasmodium yoelii*. Click here for file

Additional file 4 **Reannotated proteins with orthologs validated experimentally based on ApiLoc.** Reannotated proteins from orthologous groups previously showing mixed signal peptide prediction are shown. This information includes the status of each group after proteins reannotation, the protein reannotated from each group with their species and SP prediction status before and after annotation and the description of the orthologous proteins experimentally validated based on ApiLoc information. Species: Pb – *Plasmodium berghei*, Pf – *Plasmodium falciparum*, Pk – *Plasmodium knowlesi*, Pv – *Plasmodium vivax*, and Py – *Plasmodium yoelii*. Click here for file

Additional file 5 **Classification of orthologous groups after protein reannotations and optimization of signal peptide prediction parameters.** Classification of each orthologous group according to signal peptide prediction of their proteins in Positive (all proteins of the group showed predicted signal peptide); Negative (all proteins of the group showed prediction of absence of signal peptide); Mixed (proteins with or without predicted signal peptide in the same group). The classifications were performed before reannotation, after reannotation and after optimization of signal peptide parameters of prediction. Classification of group category after visual inspection showed groups without proteins misannotated (No misannotations); groups with all misannotated proteins corrected (Reannotated); groups with one or more proteins still misannotated (Partially reannotated). Click here for file
