# Supplementary figures and images for: Improving N-terminal protein annotation of Plasmodium species based on signal peptide prediction of orthologous proteins
Source: Malar J. 2012 Nov 15;11:375. doi: 10.1186/1475-2875-11-375 (PMC3529677; doi:10.1186/1475-2875-11-375)

No Misannotations (111 groups)

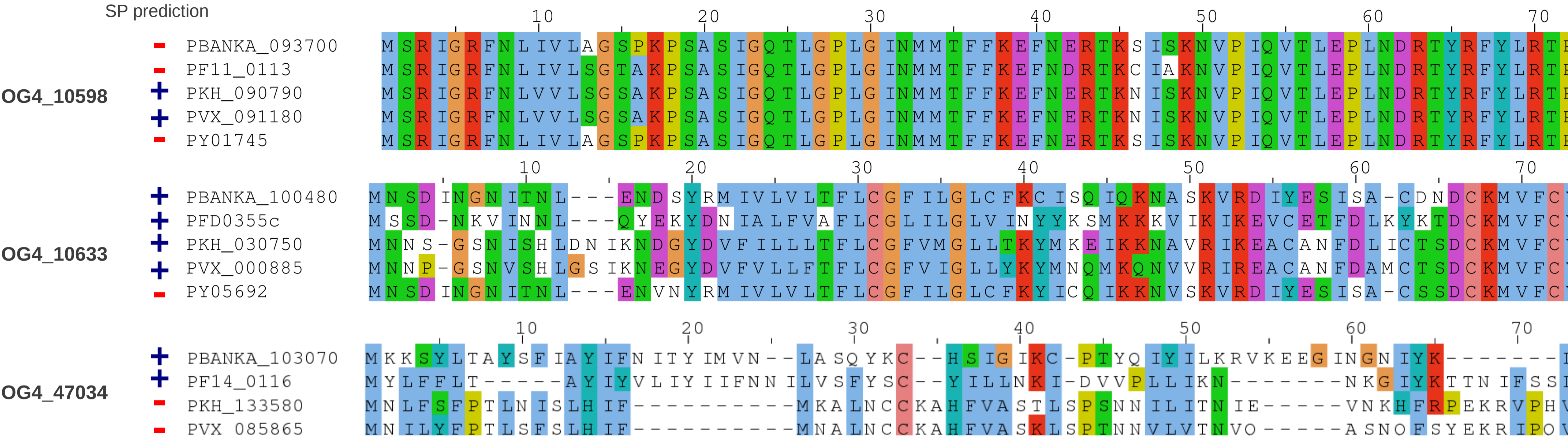

Reannotated (331 groups)

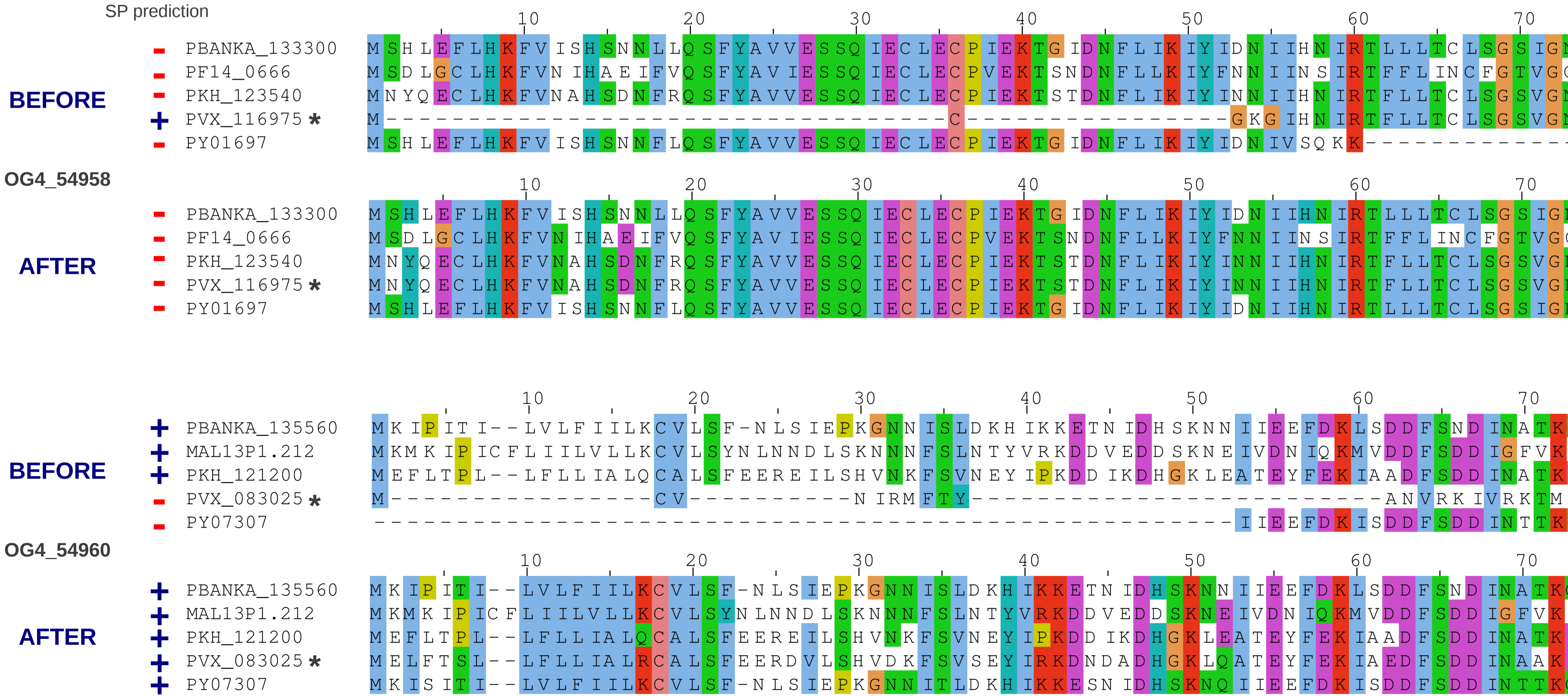

Supplement: Additional file 1 — Examples of N-terminal alignments of inspected Mixed groups. In the upper panel, three Mixed groups (OG4_10598, OG4_10633 and OG4_47034) placed in the category of No misannotations after visual inspection. Signal peptide predictions positive (+) or negative (-) are shown to the left of gene identifiers, demonstrating the Mixed nature of these groups. A total of 111 groups belong to this category. In the lower panel, two Mixed groups (OG4_54958 and OG4_54960) in which putative misannotated proteins were identified after visual inspection. Proteins in these groups were reannotated and a comparison of alignments before and after reannotations is shown with the respective signal peptide predictions to the left of gene identifiers. A total of 331 groups belong to this category (Reannotated). Reannotated P. vivax genes that were submitted to RT-PCR validation of new gene models are indicated by asterisks. [file 1475-2875-11-375-S1.pdf]
